# Supplementary material for: Human RIPK3 maintains MLKL in an inactive conformation prior to cell death by necroptosis
Source: Nat Commun. 2021 Nov 22;12:6783. doi: 10.1038/s41467-021-27032-x (PMC8608796; doi:10.1038/s41467-021-27032-x)
Supplement: Supplementary file 1 — Supplementary Information [file 41467_2021_27032_MOESM1_ESM.pdf]

SUPPLEMENTARY INFORMATION

Human RIPK3 maintains MLKL in an inactive conformation prior to cell death by necroptosis

Yanxiang Meng[1,2,4], Katherine A. Davies[1,2,4], Cheree Fitzgibbon[1,2], Samuel N. Young[1], Sarah E. Garnish[1,2], Christopher R. Horne[1,2], Cindy Luo[1], Jean-Marc Garnier[3], Lung-Yu Liang[1,2], Angus D. Cowan[1,2], Andre L. Samson[1,2], Guillaume Lessene[1,2], Jarrod J. Sandow[1,2], Peter E. Czabotar[1,2,5*], James M. Murphy[1,2,5*]

[1] Walter and Eliza Hall Institute of Medical Research, 1G Royal Parade, Parkville, VIC 3052, Australia

[2] Department of Medical Biology, University of Melbourne, Parkville, VIC 3052, Australia

[3] SYNthesis med chem, 30 Flemington Rd, Parkville, VIC 3052, Australia

[4] These authors contributed equally: Yanxiang Meng and Katherine A. Davies

[5] These authors jointly supervised the work: Peter E. Czabotar and James M. Murphy

[*]To whom correspondence should be addressed: czabotar@wehi.edu.au or jamesm@wehi.edu.au

SUPPLEMENTARY TABLES

Supplementary Table 1. SAXS data collection and analysis statistics

	**Human RIPK3:MLKL (apo)**	**Human RIPK3:MLKL (+Compound 10)**
**Data-collection parameters**		
Instrument	Australian Synchrotron SAXS/WAXS beamline	
Detector	PILATUS3-2M (Dectris)	
Wavelength (Å)	1.0332	
q range ($Å^{-1}$)[a]	0.005 to 0.5	
Detector distance (m)	3	
Maximum flux at sample	8 x 10^{12} photons/sec at 12 keV	
Temperature (K)	283	
Exposure time	Serial 1 sec exposures	
Sample configuration	SECS-SAXS with co-flow	
Flow rate	0.2 mL/min, Superdex-200 Increase 5/150 column	
Protein concentration	60 µL of 6.6 mg/ml	60 µL of 6.6 mg/ml
Inline size-exclusion chromatography buffer	20 mM HEPES pH 7.5, 0.2 M NaCl, 5% v/v glycerol	
**Structural parameters**		
I(0) (cm^{-1}) (from Guinier)	0.027 ± 0.00014	0.027 ± 0.00010
R_g (Å)[b] (from Guinier)	29.37 ± 0.25	29.39 ± 0.18
I(0) (cm^{-1}) [from P(r)]	0.027 ± 0.000085	0.027 ± 0.000077
R_g (Å) [from P(r)]	29.79 ± 0.15	29.61 ± 0.108
D_{max} (Å)	95	95
Porod volume ($Å^3$)	94,647	96,686
Molecular weight estimate (Da) (Bayesian consensus)[1]	65,450	72,400
Molecular weight from sequence (Da)	67,539.63	67,539.63
**Software employed**		
Primary data reduction	Scatterbrain (Australian Synchrotron)	
Data processing	PRIMUSQT, GNOM[2]	
Computation of model intensities	CRYSOL[3]	

[a] q is the magnitude of the scattering vector, which is related to the scattering angle (2θ) and the wavelength (λ) as follows: $q = (4\pi/\lambda)\sin\theta$

[b] R_g is the radius of gyration

Supplementary Table 2 | Oligonucleotide sequences

hRIPK3 316 stop KpnI rev‡	5´- CTCCCCCATCTCCCGTTAagaaaatctcctattgctgctcctgag-3´
NcoI RBS hRIPK3 C3S fwd	5´- GGATCTCGAGCCATGGAAACCATGtcgTCCgtcaagttatggccc-3´
pFB_HTb AccI fwd	5´-AGCTATAGTTCTAGTGGTTGGCTACGT-3´
pFB_HTb AccI rev	5´-AGAGTCCTGGGCGAACAAACG-3´
pAB2 AccI polyA fwd	5´-TTCGCCCAGGACTCTCCCCGCGTTTATGAACAAACG-3´
pAB2 p10 rev	5´-ACTAGAACTATAGCTCGGACCTTTAATTCAACCCAACACAA-3´
hMLKL 190 BamHI fwd	5´- CGCGGATCCcaagagcaaatcaaggagatcaag-3´
hMLKL 471 stop EcoRI rev	5´- CGCGAATTcacttagaaaaggtggagagtttc-3´
hMLKL T357E/S358E fwd	5´- gaggaaaacacagGAGGAaatgagtttgggaac - 3´
hMLKL T357E/S358E rev	5´- gttcccaaactcattTCCTCctgtgttttcctc - 3´
hRIPK3 2 BamHI fwd	5´- CGCGGATCCtcgtgcgtcaagttatggccc - 3´
hRIPK3 518 stop EcoRI rev	5´- CGCgaattcatttcccgctatgattatacc - 3´
hRIPK3 D142N fwd	5´- ctcctgcaccggAacctcaagccatc- 3´
hRIPK3 D142N rev	5´- gatggcttgaggtTccggtgcaggag- 3´
hRIPK3 T224A fwd	5´- gttgagttgccaGccgaaccatcac - 3´
hRIPK3 T224A rev	5´- gtgatggttcggCtggcaactcaac - 3´
hRIPK3 S227A fwd	5´- ccaaccgaaccaGcactcgtgtacg- 3´
hRIPK3 S227A rev	5´- cgtacacgagtgCtggttcggttgg - 3´

‡ Restriction sites underlined

SUPPLEMENTARY FIGURES

Supplementary Figure 1. Compound 10 binds both human RIPK3 and MLKL, but does not grossly influence complex structure.

a. Thermal stability of MLKL (black lines) and RIPK3 (red lines) in the absence (solid) or presence (dashed) of Compound 10. Duplicate assays are shown. **b-e.** *$2F_o$-Fc* electron density contoured at 1.0 σ and shown as mesh. Phosphorylation of T224 and S227 in RIPK3 (**b**) but not T357 and S358 in MLKL (**c**) was evident within the MLKL:RIPK3 complex. Density for Compound 10 was observed in the ATP-binding sites of RIPK3 (**d**) and MLKL (**e**). **f.** SAXS scattering profiles of human RIPK3:MLKL pre-incubated in the absence (apo) or in presence of 0.49 mM compound 10. Theoretical scattering profile of the human RIPK3:MLKL complex is shown as red line. χ^2 values of each experimental scattering profiles were calculated in reference to the theoretical scattering profile using CRYSOL. Guinier plots are shown as an inset. Residual plot comparing apo and compound 10 bound RIPK3:MLKL is shown on the bottom. **g.** The pairwise distribution (*P(r)*) plot calculated from the SAXS scattering profiles of human RIPK3:MLKL pre-incubated either in absence (black) or in presence of 0.49 mM Compound 10 (cyan).

Supplementary Figure 1

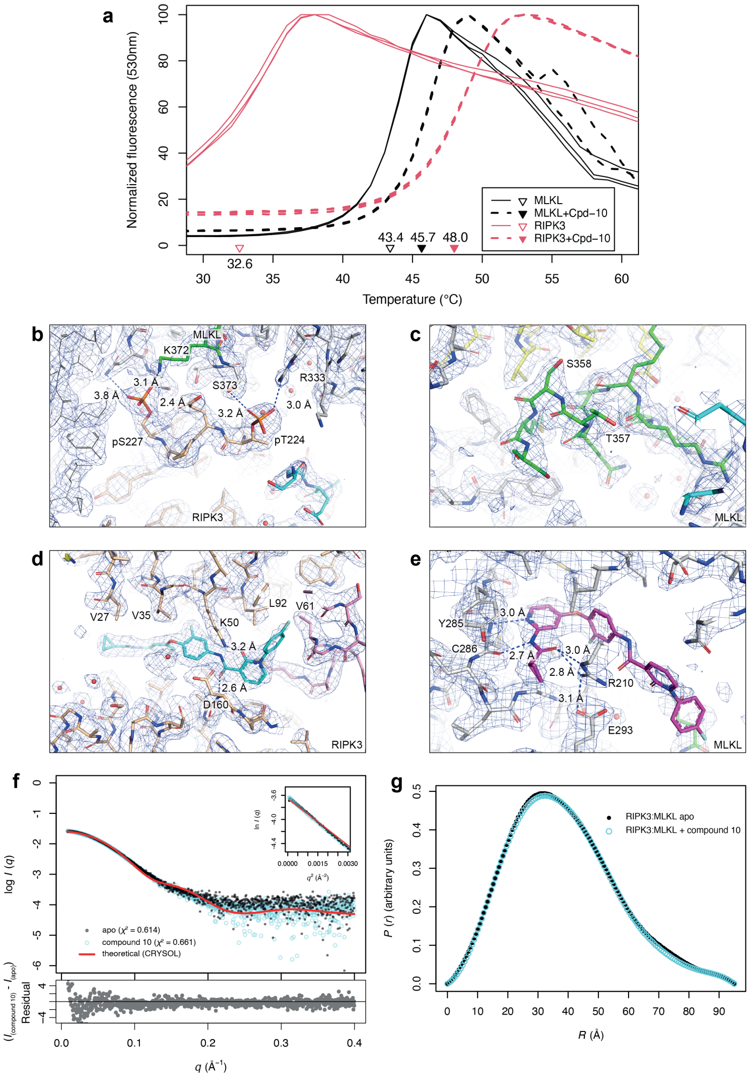

Supplementary Figure 2. Structural alignment of mouse and human MLKL and RIPK3 from their respective complexes. Related to Figures 1 and 2. a. Aligned human (top) and mouse (bottom) MLKL sequences with secondary structure elements annotated; α-helices are shown as rods, β-strands as arrows. Counterparts of catalytic motifs in protein kinases are highlighted in blue; phosphosites are highlighted in teal; and RIPK3 interacting residues as defined by PDBePISA are shown in red. **b.** Aligned human (top) and mouse (bottom) RIPK3 sequences with secondary structure elements and other features annotated as for MLKL in panel a.

Supplementary Figure 2

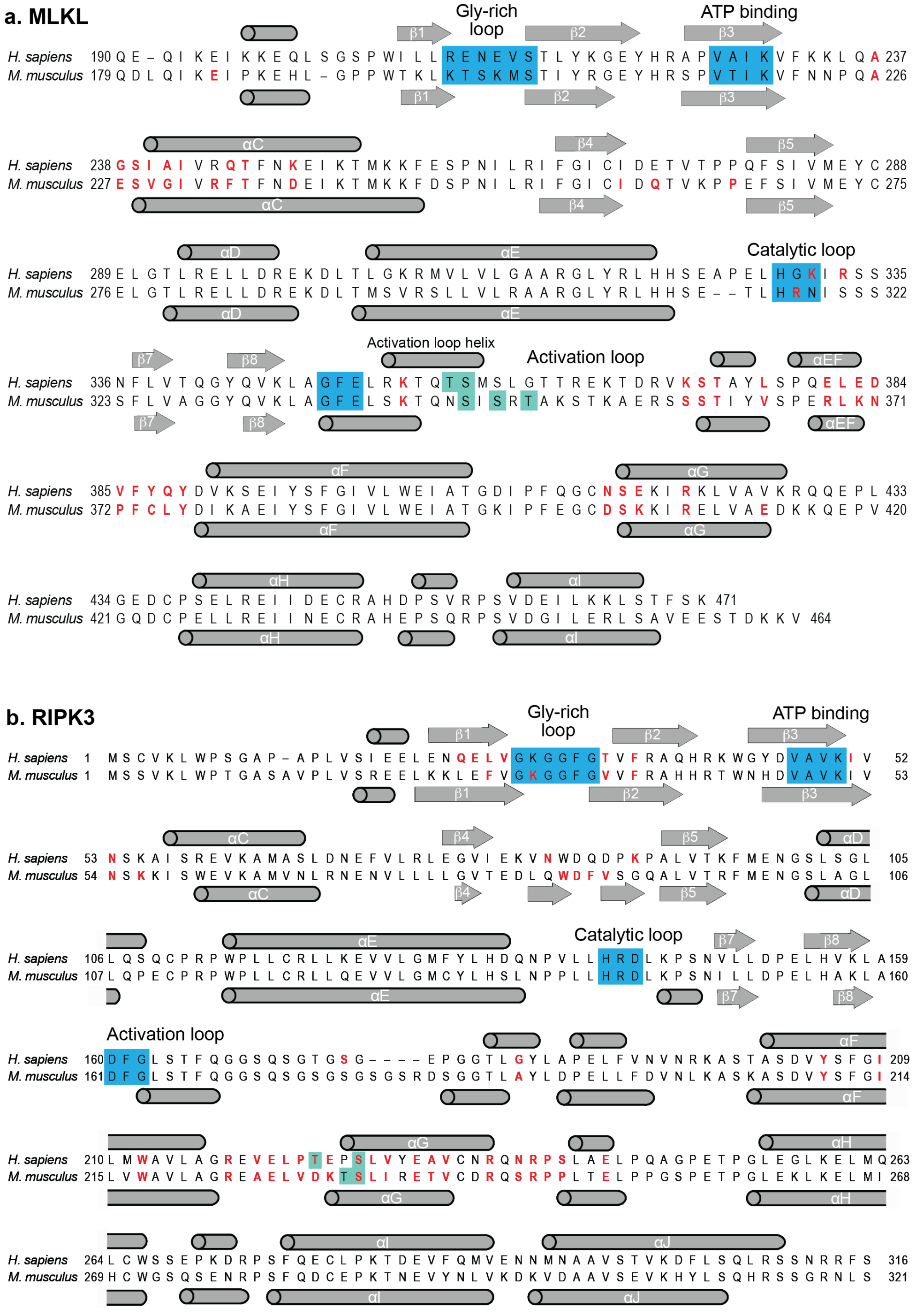

Supplementary Figure 3. Comparison of MLKL:RIPK3 interfaces in human and mouse complex structures. Superimposed human MLKL (grey):RIPK3 (magenta) (PDB: 7MON) and mouse MLKL (green):RIPK3 (blue) (PDB: 4M69)[4] complex structures. **a**. Superimposed human and mouse MLKL N-lobes. **b**. Superimposed human and mouse MLKL C-lobes. **c**. Superimposed human and mouse RIPK3 N-lobes. **d**. Superimposed human and mouse RIPK3 C-lobes. Sidechains are shown for contact residues in panels b-e, as identified using PDBePISA. **e.** In the human MLKL:RIPK3 complex, the RIPK3 substrates, MLKL T357 and S358 (activation loop, green), are distal to the catalytic Asp of RIPK3, D142 (wheat).

Supplementary Figure 3

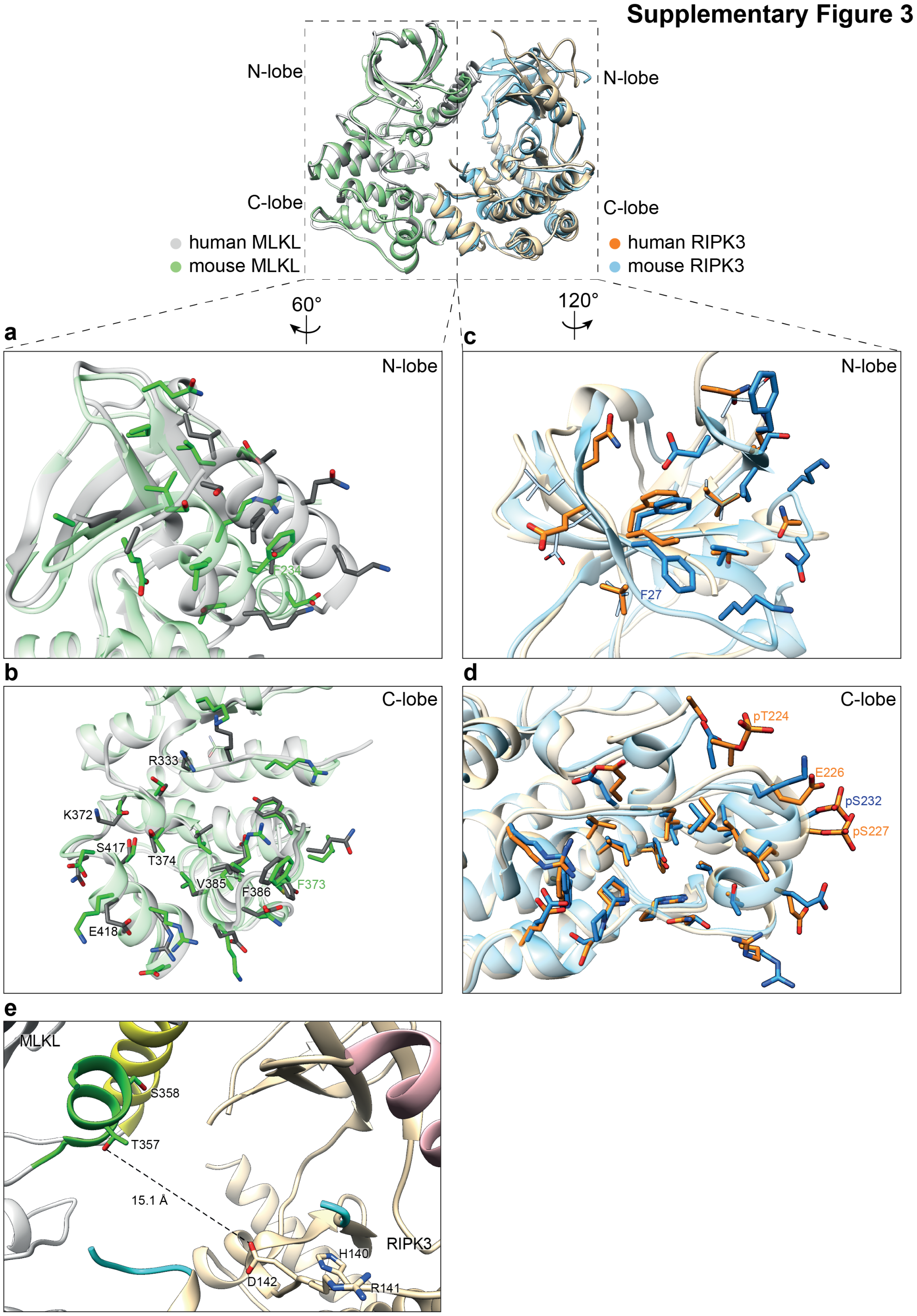

Supplementary Figure 4. Electron density maps for the human RIPK3 GSK′843 structure.
a. The αG helices and C234-C234 disulfide bond that links Chain A and C, and Chain B and D in the GSK′843-bound human RIPK3 structure. **b.** The ATP binding site of human RIPK3 in all four chains of the asymmetric unit. Residues K50 from the VAIK motif and E60 from the αC helix, which form a salt bridge, are labelled, as is the αC helix, the GSK′843 compound and F161 from the DFG motif. The blue mesh shows the σ weighted *2Fo-Fc* refined density, and the red and green mesh show the difference density map, *Fo-Fc*. Bonds are depicted as lines; purple for carbon atoms, red for oxygen, blue for nitrogen and yellow for sulfur. The *2Fo-Fc* density was contoured at 1.0 σ and *Fo-Fc* density contoured at 3.5 σ. Maps were visualised in Coot[5].

Supplementary Figure 4

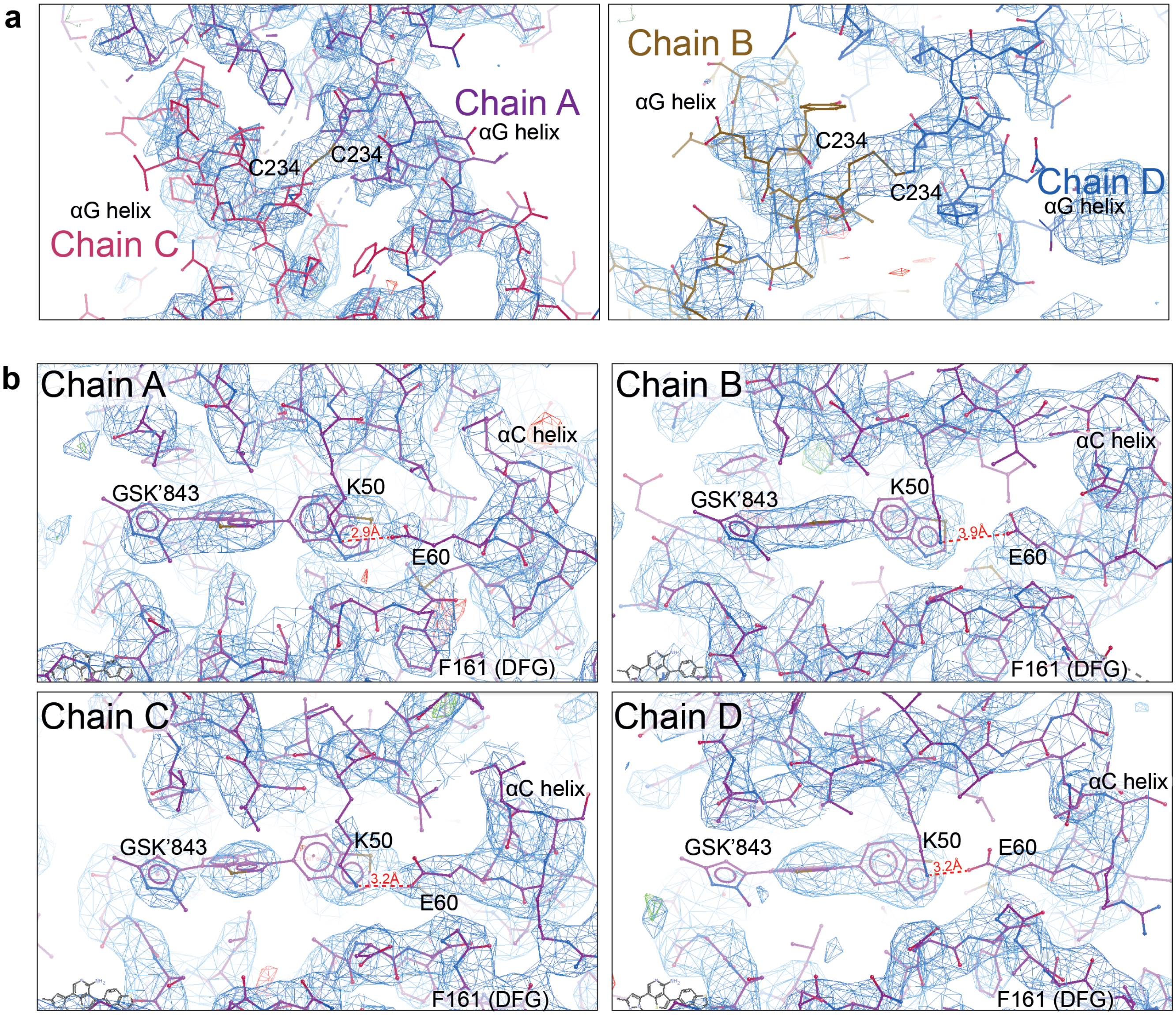

Supplementary Figure 5. Expression and necroptotic signaling of MLKL:RIPK3 interface mutants in HT29 cells.

a. Expression of wild-type and point mutant human MLKL from the RIPK3-binding interface in *MLKL*$^{-/-}$ HT29 cells upon overnight doxycycline (dox) stimulation. **b**. Expression of wild-type and point mutant human RIPK3 from the MLKL-binding interface in *RIPK3*$^{-/-}$ HT29 cells upon overnight dox stimulation. Blots in **a** and **b** are representative of 2 independent experiments. **c**. 24 hr time course monitoring cell death induced by expression of wild-type and point mutant human MLKL with and without TSI stimulation. Death measured via SYTOX Green staining detected by Incucyte. Error bars are SEM, 3 ≥ n ≤ 5 for all mutants. **d**. 24 hr time course monitoring cell death induced by expression of wild-type and point mutant human RIPK3 with and without TSI stimulation. Death measured via SYTOX Green staining detected by IncuCyte live cell imaging. Error bars are SEM, with 4 independent repeats for MLKL constructs, except n=5 for wild-type, T357E/S358E and n=3 for I242A, Q388A MLKL; and 3 independent repeats performed for all RIPK3 constructs, except n=12 for wild-type, n=8 for D142N, n=5 for E25A, L26R, F36W, L222W, L228W, N238A, N312A and n=2 for R236A RIPK3.

Supplementary Figure 5

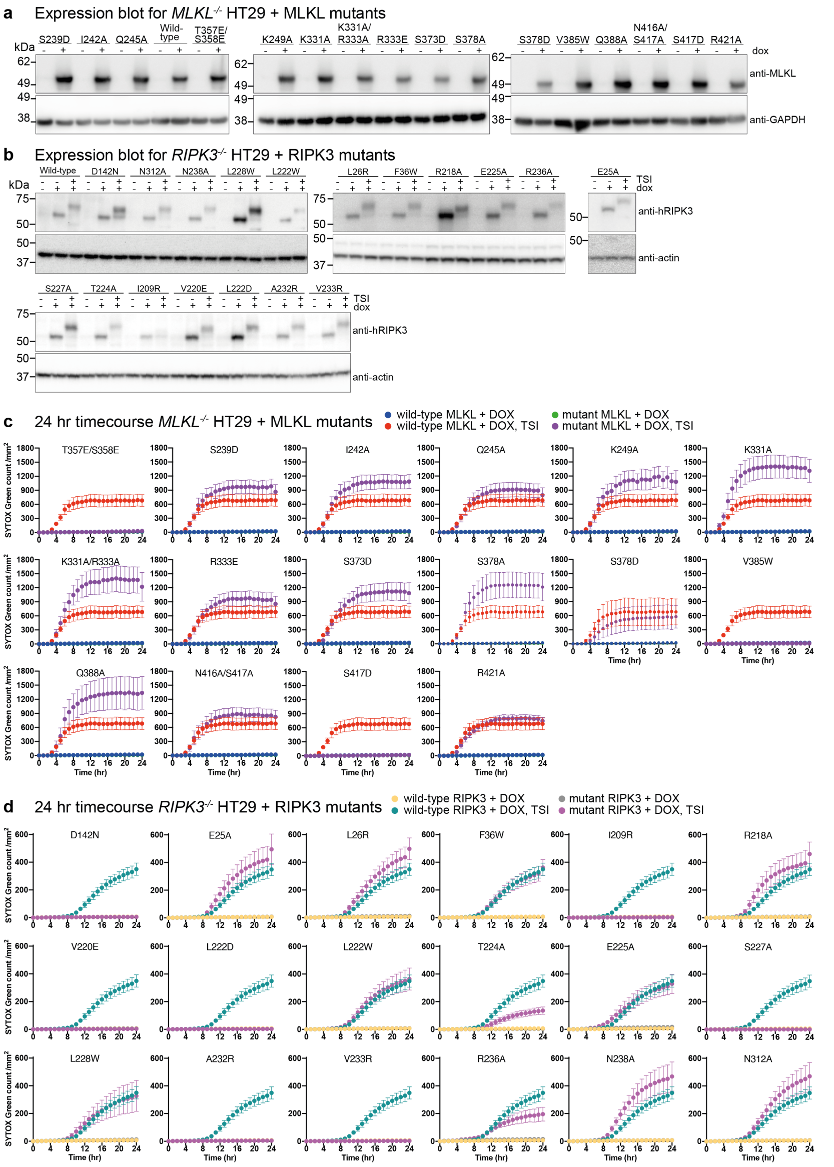

SUPPLEMENTARY REFERENCES

1. Hajizadeh NR, Franke D, Jeffries CM, Svergun DI. Consensus Bayesian assessment of protein molecular mass from solution X-ray scattering data. *Sci Rep* **8**, 7204 (2018).

2. Manalastas-Cantos K*, et al.* ATSAS 3.0: expanded functionality and new tools for small-angle scattering data analysis. *J Appl Crystallogr* **54**, 343-355 (2021).

3. Svergun DI, Barberato C, Koch MHJ. CRYSOL - a Program to Evaluate X-ray Solution Scattering of Biological Macromolecules from Atomic Coordinates. *J Appl Cryst* **28**, 768-773 (1995).

4. Xie T, Peng W, Yan C, Wu J, Gong X, Shi Y. Structural Insights into RIP3-Mediated Necroptotic Signaling. *Cell reports* **5**, 70-78 (2013).

5. Emsley P, Lohkamp B, Scott WG, Cowtan K. Features and development of Coot. *Acta Crystallogr D Biol Crystallogr* **66**, 486-501 (2010).
