# Supplementary material for: Human RIPK3 maintains MLKL in an inactive conformation prior to cell death by necroptosis
Source: Nat Commun. 2021 Nov 22;12:6783. doi: 10.1038/s41467-021-27032-x (PMC8608796; doi:10.1038/s41467-021-27032-x)
Supplement: Supplementary file 3 — Reporting summary [file 41467_2021_27032_MOESM3_ESM.pdf]

nature portfolio

Corresponding author(s): Czabotar and Murphy

Last updated by author(s): Oct 26, 2021

Reporting Summary

Nature Portfolio wishes to improve the reproducibility of the work that we publish. This form provides structure for consistency and transparency in reporting. For further information on Nature Portfolio policies, see our Editorial Policies and the Editorial Policy Checklist.

Statistics

For all statistical analyses, confirm that the following items are present in the figure legend, table legend, main text, or Methods section.

n/a	Confirmed	
☐	☒	The exact sample size (*n*) for each experimental group/condition, given as a discrete number and unit of measurement
☐	☒	A statement on whether measurements were taken from distinct samples or whether the same sample was measured repeatedly
☒	☐	The statistical test(s) used AND whether they are one- or two-sided<br>*Only common tests should be described solely by name; describe more complex techniques in the Methods section.*
☒	☐	A description of all covariates tested
☒	☐	A description of any assumptions or corrections, such as tests of normality and adjustment for multiple comparisons
☐	☒	A full description of the statistical parameters including central tendency (e.g. means) or other basic estimates (e.g. regression coefficient) AND variation (e.g. standard deviation) or associated estimates of uncertainty (e.g. confidence intervals)
☐	☒	For null hypothesis testing, the test statistic (e.g. *F*, *t*, *r*) with confidence intervals, effect sizes, degrees of freedom and *P* value noted<br>*Give P values as exact values whenever suitable.*
☒	☐	For Bayesian analysis, information on the choice of priors and Markov chain Monte Carlo settings
☒	☐	For hierarchical and complex designs, identification of the appropriate level for tests and full reporting of outcomes
☒	☐	Estimates of effect sizes (e.g. Cohen's *d*, Pearson's *r*), indicating how they were calculated

Our web collection on statistics for biologists contains articles on many of the points above.

Software and code

Policy information about availability of computer code

Data collection	Crystallographic data were collected using MX controls (Australian Synchrotron MX beamline control software; in-house, no versioning). ScatterBrain v2.82 (Australian Synchrotron) was used to radially average, normalize against sample transmission, and background-subtract SAXS data. Incucyte S3 data were collected on an instrument with firmware version 20202.2.0.0.
Data analysis	Phenix: 1.18.2, Coot: 0.9 EL, AutoPROC: 1.0.5, DIALS: 2.2.0 were used for crystallographic data processing, model building and refinement.<br>UCSF Chimera 1.14 and PyMol 2.3.4 were used for structure analyses and depiction.<br>ATSAS-3.0.2-0 was used for SAXS analyses.<br>Incucyte 2020C Rev1 software was used to analyse live cell imaging data.

For manuscripts utilizing custom algorithms or software that are central to the research but not yet described in published literature, software must be made available to editors and reviewers. We strongly encourage code deposition in a community repository (e.g. GitHub). See the Nature Portfolio guidelines for submitting code & software for further information.

Data

Policy information about availability of data

All manuscripts must include a data availability statement. This statement should provide the following information, where applicable:
- Accession codes, unique identifiers, or web links for publicly available datasets
- A description of any restrictions on data availability
- For clinical datasets or third party data, please ensure that the statement adheres to our policy

All data, including expression construct sequences, are available from the corresponding authors upon request. The atomic coordinates for the human RIPK3 kinase domain in complex with GSK843, and in complex with the human MLKL pseudokinase domain and Compound 10, have been deposited in the Protein Data Bank with

the accession numbers, 7MX3 [http://doi.org/10.2210/pdb7MX3/pdb] and 7MON [http://doi.org/10.2210/pdb7MON/pdb], respectively. The atomic coordinates for previously reported structures are available from the Protein Data Bank with accession codes: 4MWI, 7JXU, 7JW7, 4M66, 4M69, 6OKO, 3CE3, 6BWK.

Field-specific reporting

Please select the one below that is the best fit for your research. If you are not sure, read the appropriate sections before making your selection.

[x] Life sciences [ ] Behavioural & social sciences [ ] Ecological, evolutionary & environmental sciences

For a reference copy of the document with all sections, see nature.com/documents/nr-reporting-summary-flat.pdf

Life sciences study design

All studies must disclose on these points even when the disclosure is negative.

Sample size	All cell-based experiments were performed typically with singlicate human cell lines, as noted in methods and legends. The numbers of independent repeat experiments are stated in figure legends. Because our prior studies (e.g. Petrie et al., Nature Commun 2018; Petrie et al., PNAS 2020; Garnish et al., Nature Commun 2021) have shown robust consistency between assays, n of 3 or more were established as sufficient to reveal any differences between cell lines.
Data exclusions	All data are presented except in cases where a technical reason compromised the experiment, such as when high background cell death was observed in control conditions or when IncuCyte measurements were compromised, such as by a speck of dust affecting the microscopy. The omitted datasets are included in source data, along with explanations for their omission.
Replication	Experiments were repeated independently typically at least in triplicate, as stated in legends, except some experiments that were performed in duplicate as stated. The natural spread of the data is shown in the paper through use of individual data points and standard error of the mean error bars. All attempts at replication were successful.
Randomization	This is not relevant to our study. Our study compared the death of cell lines expressing different exogenes upon stimulation in parallel, or the characterization of different purified proteins in parallel. The order by which mutants were examined differed between experiments based on the availability of sufficient cultured cells for assays. Therefore the experiments were performed in different orders for each repeat, but were not formally randomized.
Blinding	This is not relevant to our study. Our study compared the death of cell lines expressing different exogenes upon stimulation in parallel, or the binding activities of different purified proteins in parallel. All cell death and binding experiments were performed with no a priori assumptions of residue involvement in binding beyond their presence in the structure interface, and as such each experiment sought to establish their relative contributions and did not necessitate blinding. Controls were run in parallel with mutant constructs; to achieve this blinding was not possible.

Reporting for specific materials, systems and methods

We require information from authors about some types of materials, experimental systems and methods used in many studies. Here, indicate whether each material, system or method listed is relevant to your study. If you are not sure if a list item applies to your research, read the appropriate section before selecting a response.

Materials & experimental systems

n/a	Involved in the study	
[ ]	[x]	Antibodies
[ ]	[x]	Eukaryotic cell lines
[x]	[ ]	Palaeontology and archaeology
[x]	[ ]	Animals and other organisms
[x]	[ ]	Human research participants
[x]	[ ]	Clinical data
[x]	[ ]	Dual use research of concern

Methods

n/a	Involved in the study	
[x]	[ ]	ChIP-seq
[x]	[ ]	Flow cytometry
[x]	[ ]	MRI-based neuroimaging

Antibodies

Antibodies used	Primary antibodies used in this study were: rat anti-MLKL (clone 3H1, produced in-house; 1:1000 dilution; available as MABC604, EMD Millipore, Billerica, MA, USA), rat anti-human MLKL pseudokinase domain (clone 7G2, produced in-house; 1:2000 dilution), rabbit anti- human MLKL phospho-S358 (AB187091, Abcam; 1:3000), mouse anti-Actin (C4) HRP (sc-4778 HRP, Santa Cruz Biotechnology; 1:10000), rat anti-human RIPK3 (clone 1H2, produced in-house; 1:1000 dilution; available from Millipore as MBC1640), rabbit anti- human phospho-S227 (D6W2T, CST, 1:2000).
Validation	MLKL, pMLKL, RIPK3, pRIPK3 antibodies have been validated for selectivity using KO cell lines in Samson et al. Cell Death Differ (2021). Anti-Actin has been validated by the manufacturer (https://datasheets.scbt.com/sc-47778.pdf).

Eukaryotic cell lines

Policy information about cell lines

Cell line source(s)	HT29 cells were supplied by Mark Hampton and originate from ATCC. CRISPR-edited derivative HT29 cells lacking MLKL were reported previously (Petrie et al., Nature Commun 2018) while RIPK3-deficient HT29 cells were described recently (Garnish et al., Nature Commun 2021). Sf21 cells were used for insect cell expression (purchased from Thermo).
Authentication	None of the cells were formally authenticated. However, their morphologies are consistent with the stated cell type. The lack of responsiveness of MLKL- or RIPK3-deficient human HT29 cells to necroptotic stimuli and the lack of respective MLKL or RIPK3 reactivity by western blot are consistent with the absence of MLKL or RIPK3. Sf21 cells were susceptible to baculovirus infection and produced recombinant proteins encoded by these baculoviruses, consistent with their authenticity.
Mycoplasma contamination	All mammalian lines used were monitored for mycoplasma and were found to be negative in our routine screening. Sf21 insect cells were not monitored for mycoplasma contamination.
Commonly misidentified lines (See ICLAC register)	Nil
