# Supplementary material for: Human RIPK3 maintains MLKL in an inactive conformation prior to cell death by necroptosis
Source: Nat Commun. 2021 Nov 22;12:6783. doi: 10.1038/s41467-021-27032-x (PMC8608796; doi:10.1038/s41467-021-27032-x)

Uncropped western blots for Supplementary Figure 5a

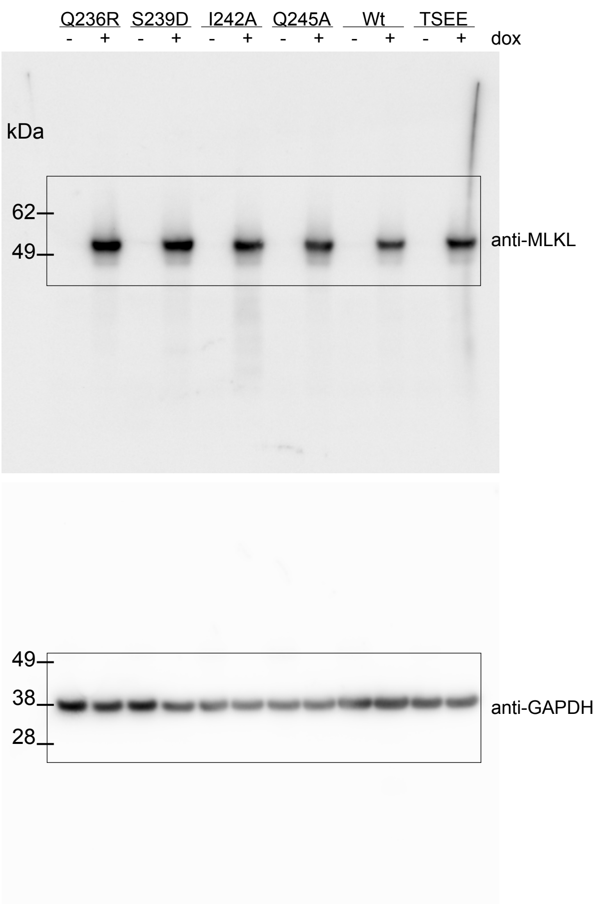

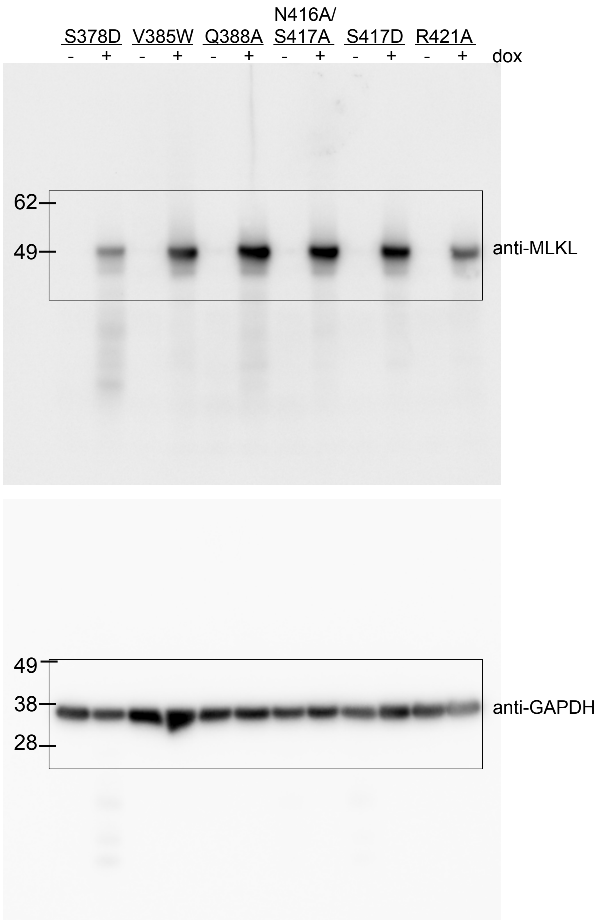

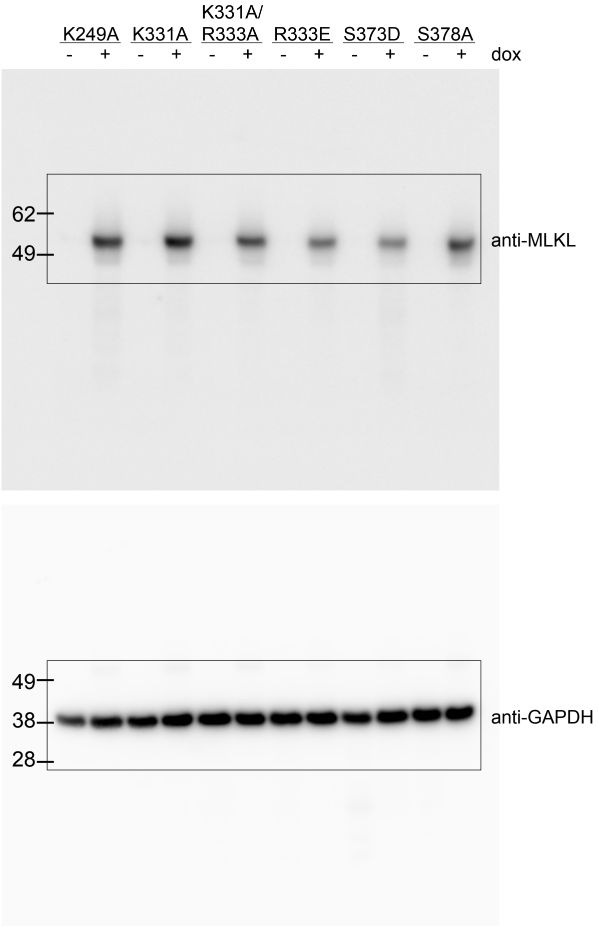

Uncropped western blots for Supplementary Figure 5b

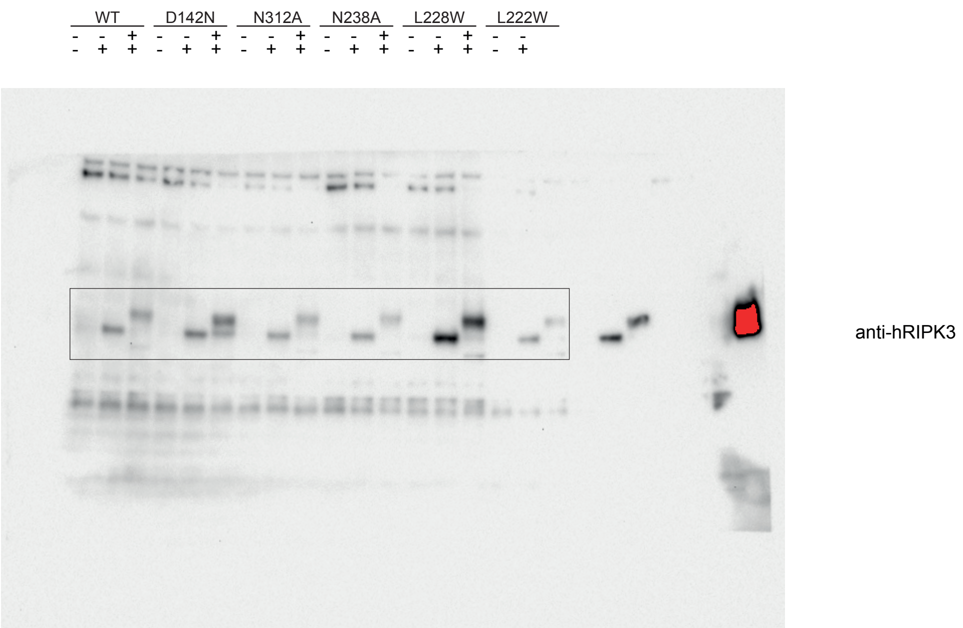

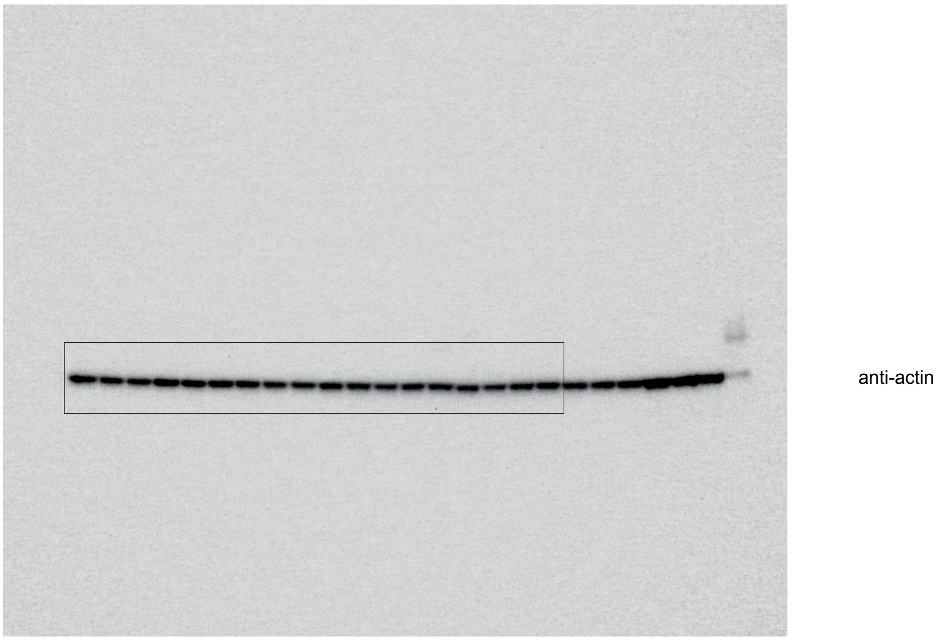

Uncropped western blots for Supplementary Figure 5b

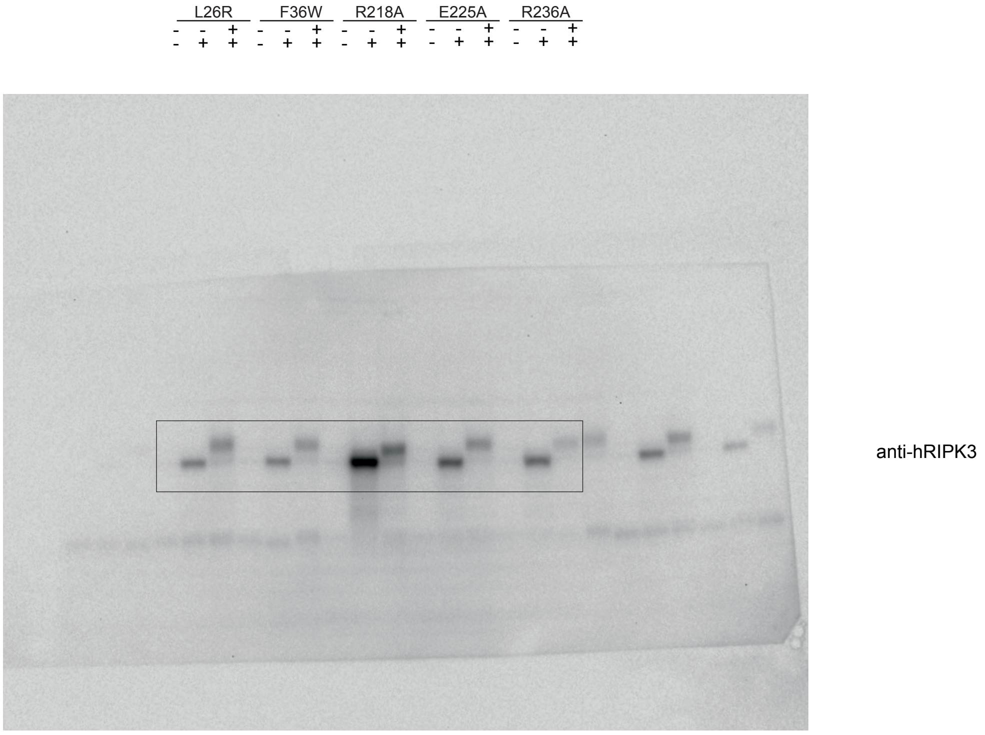

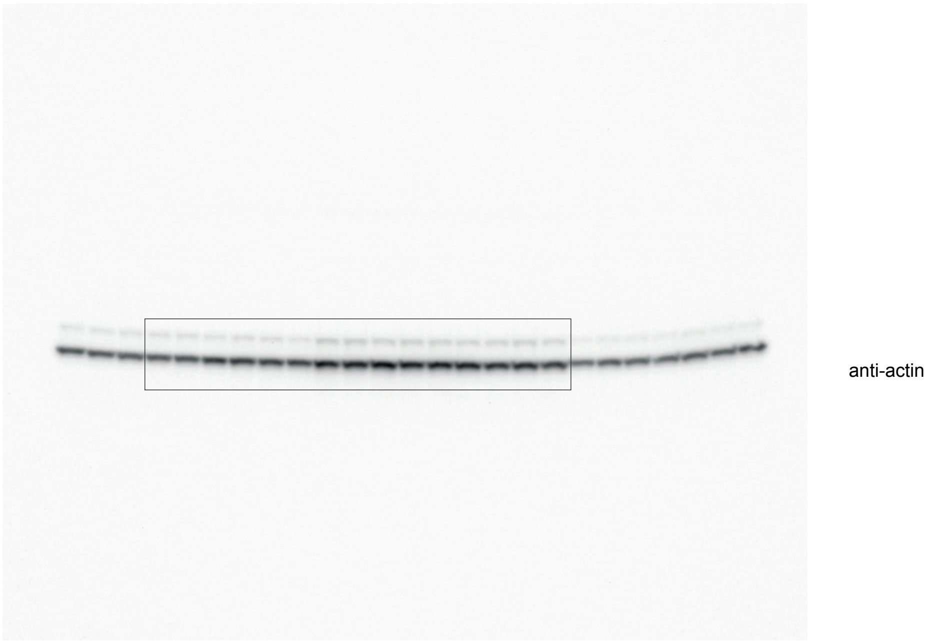

Uncropped western blots for Supplementary Figure 5b

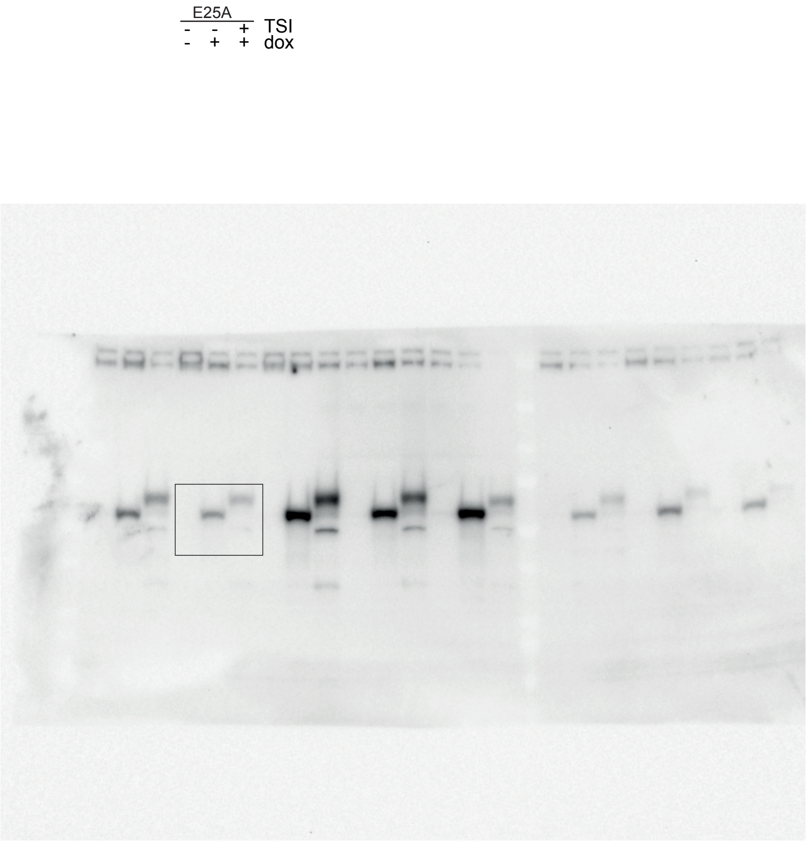

anti-hRIPK3

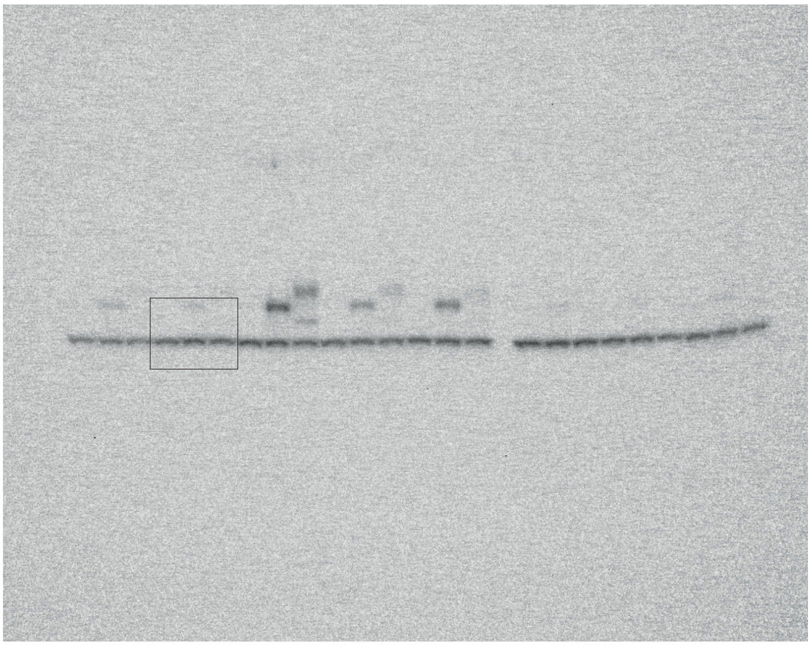

anti-actin

Uncropped western blots for Supplementary Figure 5b

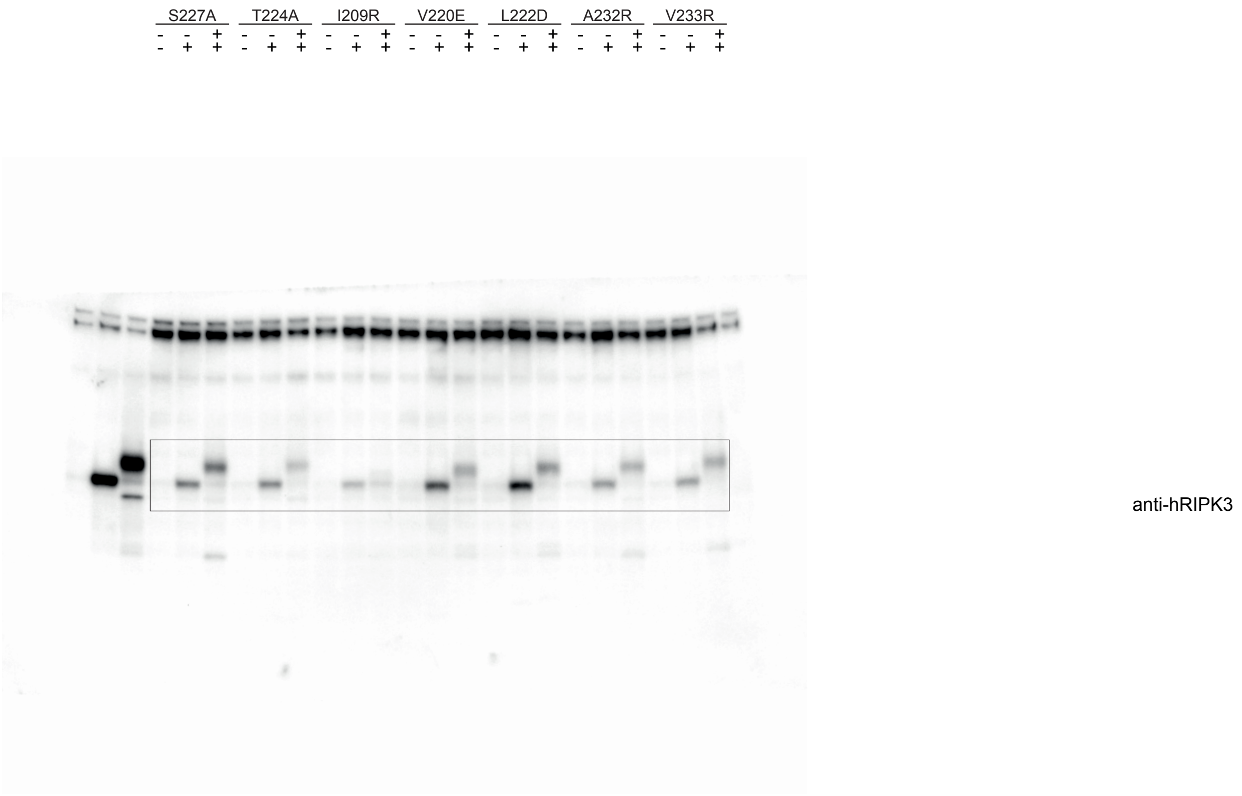

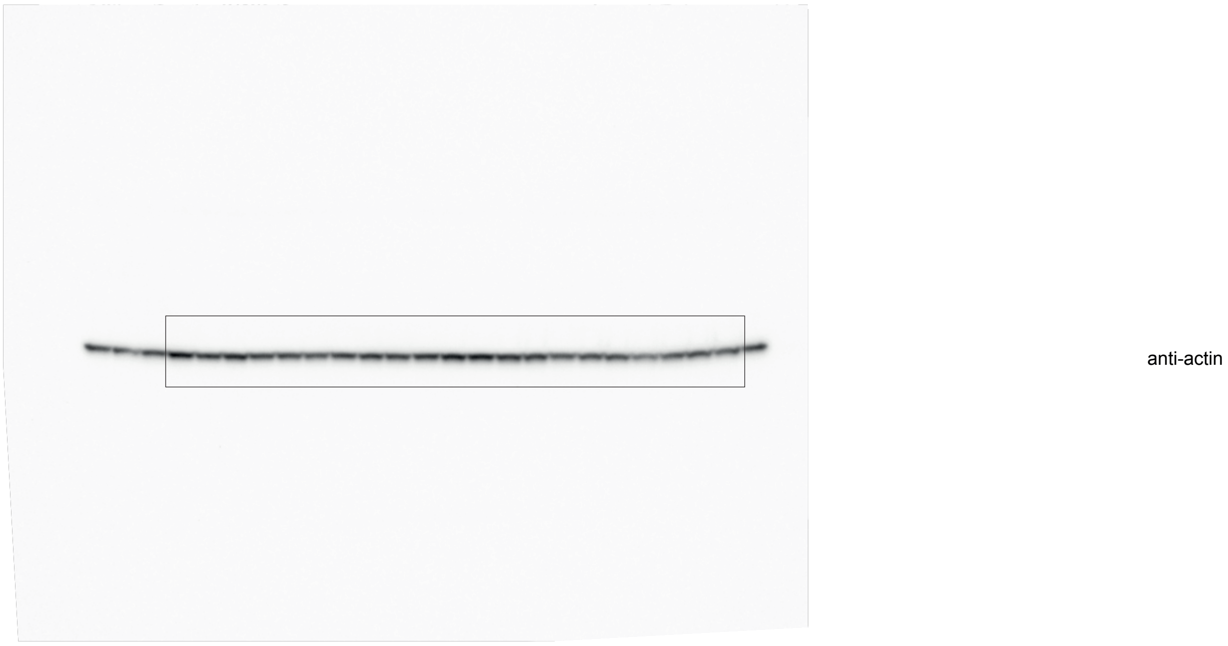

Supplement: Supplementary file 4 — Source Data [file 41467_2021_27032_MOESM4_ESM.zip › Source data/Source Supp Figure 5 blots_130921.pdf]
